# Emergency Department Utilization Patterns Among Physical Elder Abuse Victims in Comparison to Other Older Adults

**DOI:** 10.1093/geroni/igab046.579

**Published:** 2021-12-17

**Authors:** Tony Rosen, Katherine Wen, Sunday Clark, Alyssa Elman, Philip Jeng, Yiye Zhang, Karl Pillemer, Yuhua Bao

**Affiliations:** 1 Weill Cornell Medical College / NewYork-Presbyterian Hospital, PELHAM, New York, United States; 2 Cornell University, Ithaca, New York, United States; 3 Boston University School of Medicine, Boston, Massachusetts, United States; 4 Weill Cornell Medical College / NewYork-Presbyterian Hospital, New York, New York, United States; 5 Weill Cornell Medical College, New York, New York, United States

## Abstract

Background: Physical elder abuse is common and has serious health consequences. Little is known, however, about the patterns of health care utilization among these victims, including whether opportunities may exist for earlier identification and intervention. Our goal was to describe Emergency Department (ED) utilization known physical elder abuse victims compared with non-victims. Methods: We used Medicare insurance claims to examine ED utilization patterns among a well-characterized cohort of 139 known physical elder abuse victims in the year before abuse was identified and compared this to control subjects matched on age, sex, race, and residential zip code. Results: Physical elder abuse victims were significantly more likely than control subjects to visit the ED (47.5% vs. 35.9%, p=0.01) during the year before identification and to have at least one visit for an injury-related complaint (14.4% vs. 8.3%, p=0.03). Victims were also more likely to have multiple visits (18.7% vs. 14.6%, p=0.24), visit multiple EDs (7.9% vs. 6.7%, p=0.63), or be high frequency utilizers (≥4 visits, 3.6% vs. 2.7%, p=0.58), but differences did not reach statistical significance. The most common diagnoses in ED visits among victims were: open wound of knee/ankle, exacerbation of chronic bronchitis, pneumonia, and chest pain. Conclusion: This work provides preliminary evidence that physical elder abuse victims use the ED more frequently and potentially have different patterns of utilization than other older adults. We plan to further characterize these different patterns to potentially to use them to develop tools for earlier identification.

